# Ultrasound characterization of superficial lymph nodes in HIV patients with *Talaromyces marneffei* infection

**DOI:** 10.3389/fmed.2023.1243599

**Published:** 2023-12-11

**Authors:** Lin Pan, Huaguo Shao

**Affiliations:** ^1^Department of Ultrasound, Affiliated Hangzhou Xixi Hospital, Zhejiang University School of Medicine, Hangzhou, China; ^2^Institute of Hepatology and Epidemiology, Affiliated Hangzhou Xixi Hospital, Zhejiang University School of Medicine, Hangzhou, China

**Keywords:** HIV, *Talaromyces marneffei*, ultrasound, lymph node, diagnosis

## Abstract

**Objectives:**

This study aimed at exploring the ultrasound characteristics of superficial lymph nodes (LNs) in HIV patients with *Talaromyces marneffei* infection to provide assistance and understanding for diagnosis and therapy.

**Methods:**

A retrospective analysis was conducted on 26 patients with confirmed HIV and *T.marneffei* coinfection. These patients underwent ultrasound examination and ultrasound-guided puncture biopsies at our hospital from March 2015 to March 2023.

**Results:**

In all 26 patients, lymphadenectasis was observed. Among the 21 cases (80.76%), LNs showed a diffusely hyperechoic appearance with a tulle-like change, and 6 cases (23.07%) showed liquefaction. When the hila were present or thinned, the blood flow signals were primarily hilar, whether rich or poor, and when the hila were absent, the blood flow signals were peripheral or poor. The axillary LN long-to-short diameter (L/S) ratios exhibited a significant positive correlation with CD4^+^T cell counts (*r* = 0.8214, *p* = 0.0341). Patients with retroperitoneal lymphadenectasis showed decreased NK cell counts (*p* = 0.03).

**Conclusion:**

In summary, the *T.marneffei* infection of LNs in HIV patients often manifests with superficial LN enlargement, mostly affecting the cervical LNs. The *T.marneffei*-infected LNs exhibit several characteristics such as echogenicity, hilum, and blood flow signal. Furthermore, there might be associations between lymphocyte subsets and enlarged superficial LNs. Ultrasound examinations should be paid attention to if patients have superficial LN enlargement, and the diagnosis of the *T.marneffei* infection is considered.

## 1 Introduction

Human immunodeficiency virus (HIV) is a retrovirus that causes acquired immune deficiency syndrome (AIDS) (1). HIV destroys and disables the immune system, often leading patients to die from secondary infections and cancer (2). According to the Joint United Nations Program on HIV and AIDS (UNAIDS), 38 million people were living with HIV worldwide in 2019 (3). The Chinese Center for Disease Control and Prevention (CCDC) estimated that, in China, there were more than 0.86 million people living with HIV at the end of 2018, with 83.4% of them receiving anti-retroviral therapy (ART) (4, 5).

Upon acquiring the infection, HIV specifically attacks CD4^+^T lymphocytes in humans, resulting in the decrease or dysfunction of CD4^+^T lymphocytes. This leads to secondary infection or secondary tumors due to immune deficiency syndrome (6). Among secondary infections, fungal infection causes at least a quarter of deaths in people living with HIV (PLWH) (7). *Talaromyces marneffei* (*Penicillium marneffei*) is one of the major causes of opportunistic fungal infection in HIV-related secondary infections prevalent in Southeast and East Asia, with the highest incidence in China, Thailand, and Vietnam (8, 9). Diagnosis can be inferred from fungal culture and microscopic examination (10). Patients with lower CD4^+^T lymphocytes are more likely to experience severe clinical manifestations (11, 12). When infected, patients usually develop papulonecrotic skin lesions, fever, lymphadenectasis, and gastrointestinal abnormalities, often accompanied by other opportunistic infections such as tuberculosis and salmonella infections (13, 14). Even with ART treatment, the mortality rate of the *T.marneffei* infection can still reach 33% in China (15). Given the high fatality rate, timely diagnosis and treatment are crucial for HIV patients (16). In the current times, the most accurate diagnosis is the microscopic identification of morphological and dimorphic fungi separated and cultured from clinical specimens (17). However, the fungal culture needs to be incubated for at most 2 weeks, resulting in an elevated case fatality rate (18).

Ultrasound examination uses ultrasound microwaves to image the organs and tissues inside patients. Unlike computed tomography (CT), which produces ionizing radiation, ultrasound examinations do not harm the human body. For *T.marneffei*-infected patients, ultrasound examination can be used to investigate changes in lymph node (LN) lesions. While there are several case reports and clinical studies on the manifestation and CT results of HIV patients with *T.marneffei* (19–22), studies on ultrasound imaging of the *T.marneffei* infection are rarely reported. Based on the studies cited above, we speculate that there are some distinguishable sonographic features and a potential relationship between these features and the immune status of these patients. Therefore, the objectives of this study were to retrospectively characterize the ultrasound features of superficial LNs and explore the associations between lymphocyte subsets and sonographic features in HIV patients infected with *T.marneffei* to provide assistance and understanding for diagnosis and therapy.

## 2 Materials and methods

### 2.1 Patients

This retrospective analysis was performed on 26 HIV patients with *T.marneffei* infection, who underwent ultrasound examination at the Affiliated Hangzhou Xixi Hospital of Zhejiang University School of Medicine, Hangzhou, Zhejiang, China from March 2015 to March 2023. The diagnostic criteria for HIV were based on the Chinese guidelines for the diagnosis and treatment of HIV/AIDS in 2021 (23). All patients were confirmed to be infected with *T.marneffei* through ultrasound-guided needle puncture biopsy of superficial LNs, with the tissue culture demonstrating morphological similarity to *T.marneffei* under a microscope (Supplementary Figure S1). Biopsy specimens were collected from right cervical LNs, left axillary LNs, and left cervical LNs in 2, 3, and 21 patients, respectively. The latest measurement of lymphocyte subsets before the superficial LN biopsy of each patient was also obtained.

### 2.2 Ultrasound examination

Ultrasound examinations of LNs were performed using LOGIQ E9 (GE) and EPIQ5 (PHILIPS) diagnostic ultrasound systems equipped with a 6–15 MHz linear-array probe. The cervical, axillary, and inguinal areas of patients were scanned routinely, and the distribution, number, size, shape, long diameter, short diameter, echogenicity, and blood flow signal of infected LNs were observed. Real-time image data were recorded during the examination. The LN L/S ratio was calculated by dividing the long diameter of the LN by the short diameter. All ultrasound examination data were analyzed by at least one associate chief physician and one chief physician with more than 5 years of experience in the ultrasound department.

### 2.3 Statistical analysis

All statistical analyses were conducted using R 4.2.2 software. Given the small sample size, the observation values were described as the median with interquartile range (IQR) and analyzed using the Mann–Whitney U non-parametric test. Quantitative data were reported by frequency. The Spearman rank correlation test was used for correlation analysis. A value of *p* of <0.05 was considered statistically significant for all test analyses in this study.

## 3 Results

### 3.1 Lymphocyte subsets

Examination results for HIV RNA viral loads and lymphocyte subsets were collected from 26 patients, but one result was missing (Table 1).

**Table 1:** Lymphocyte subsets in HIV patients infected with TM.

Parameter	Value	Cases (%)	Reference range
Sex	Male	26 (100)	—
HIV RNA viral loads	>100	18 (72)	—
	Undetectable	7 (28)	
CD4^+^T cell count	17 (36)	25 (96.15)	250–1561.6
CD8^+^T cell count	241 (216)	25 (96.15)	203.5–1347.2
B cell count	24 (64)	25 (96.15)	77–736
NK cell count	44 (97)	25 (96.15)	88–640
T cell count	292 (229)	25 (96.15)	660–2,720
Lymphocyte count	370 (440)	25 (96.15)	1,100–3,200
CD4^+^T cell ratios	4.57 (8.94)	26 (100)	24.5–48.8
CD8^+^T cell ratios	59.51 (21.73)	26 (100)	18.5–42.1
CD4^+^T/CD8^+^T cell ratios	11.39 (13.26)	26 (100)	1.2–1.94
B cell ratios	9.12 (9.83)	26 (100)	7–23
NK cell ratios	13.24 (15.06)	26 (100)	8–20
T cell ratios	74.06 (24.45)	26 (100)	60–85

All patients included in this study belonged to male sex (100%). HIV RNA viral loads were more than 100 (copies/ml) in 18 patients (72%), undetectable in 7 patients (28%), and negative in 2 patients (7.69%). Compared to the reference range, CD4^+^T cell counts were decreased in all patients (100%, median: 17 cells/μL, IQR: 36 cells/μL, range: 1–225 cells/μL), with less than 200, 100, and 50 cells/μL in 23 (92%), 21 (84%), and 20 (80%) patients, respectively. CD4^+^T cell ratios were also all less than the reference range (100%, median: 4.57%, IQR: 8.94%, range: 0.31–13.69%). CD8^+^T cell counts were decreased in 10 patients (40%, median: 241 cells/μL, IQR: 216 cells/μL, range: 41–1,046 cells/μL), but CD8^+^T cell ratios were increased in 23 patients (88.46%, median 59.51%, IQR 21.73%, range: 31.56–84.47%). B cell counts of 19 patients (76%, median: 24 cells/μL, IQR: 64 cells/μL, range: 3–196 cells/μL) were decreased, and B cell ratios of 10 patients (38.46%, median: 9.12%, IQR: 9.83%, range: 0.81–48.44%) were decreased. NK cell counts were decreased in 17 patients (68%, median: 44 cells/μL, IQR: 97 cells/μL, range: 88-640 cells/μL). NK cell ratios were increased in 10 patients (38.46%, median: 13.24%, IQR: 15.06%, range: 4.32–40.2%) and decreased in 5 patients (19.23%). T cell counts were decreased in 22 patients (88%, median: 292 cells/μL, IQR: 229 cells/μL, range: 61–1,318 cells/μL). T cell ratios were increased in three patients (11.54%, median: 74.06%, IQR: 24.45%, range: 43.57–94.86%) and decreased in seven patients (26.92%). Lymphocyte counts of 22 patients (88%, median: 370 cells/μL, IQR: 440 cells/μL, range: 100–1720 cells/μL) were lower than the reference range.

### 3.2 Sonographic features

Sonographic features of cervical LNs, axillary LNs, inguinal LNs, and retroperitoneal LNs for 26 patients are listed in Table 2.

**Table 2:** Ultrasound findings in HIV patients infected with TM.

Parameter	Value		Cases (%)
Hepatomegaly	No		15 (57.69)
	Yes		11 (42.31)
Splenomegaly	No		13 (50)
	Yes		13 (50)
Ascites	No		20 (76.92)
	Mild		5 (19.23)
	Moderate		1 (3.85)
Cervical lymphadenectasis	No		2 (7.69)
	Sinistral multiple		1 (3.85)
	Bilateral multiple		23 (88.46)
Cervical LN L/S ratio	2 (0.74)		24 (92.31)
Axillary lymphadenectasis	No		19 (73.08)
	Sinistral multiple		2 (7.69)
	Dextral multiple		1 (3.85)
	Bilateral multiple		4 (15.38)
Axillary LN L/S ratio	1.86 (1.11)		7 (26.92)
Inguinal lymphadenectasis	No		21 (80.77)
	Dextral multiple		1 (3.85)
	Bilateral multiple		4 (15.38)
Inguinal LN L/S ratio	3.5 (1.13)		5 (19.23)
Retroperitoneal lymphadenectasis	No		14 (53.85)
	Multiple		12 (46.15)
Retroperitoneal LNs L/S ratio	1.92 (0.55)		5 (19.23)
Shape	Regular		26 (100)
Margin	Defined		26 (100)
Echogenicity	Hypoechoic	Heterogeneous	1 (3.85)
		Homogeneous	4 (15.38)
	Hyperechoic	Heterogeneous	6 (23.08)
		Homogeneous	15 (57.69)
Hilum of LNs	Absent		12 (46.15)
	Thinned		10 (38.46)
	Present		4 (15.38)
Blood flow signal	No		2 (7.69)
	Mixed	Poor	1 (3.85)
	Hilar	Poor	6 (23.08)
		Rich	7 (26.92)
	Peripheral	Poor	8 (30.77)
		Rich	2 (7.69)
Fusion	No		1 (3.85)
	Partial		25 (96.15)
Calcification	No		26 (100)
Liquefaction	No		20 (76.92)
	Partial		4 (15.38)
	Most		2 (7.69)

Most of the lesions of LNs in *T.marneffei* had multiple enlargements, defined margins (100%), and regular shapes (100%). As the most commonly involved LNs, cervical lymphadenectasis occurred in 24 patients (92.31%), with 1 case (3.85%) being sinistral multiple and 23 cases (88.46%) being bilateral multiple. Cervical LN L/S ratios were less than or equal to 2 in 13 patients (54.17%, median: 2, IQR: 0.74, range: 1.2–4). Additionally, multiple regions were involved. Axillary lymphadenectasis occurred in seven cases (26.92%), with two (7.69%), one (3.85%), and four (15.38%) cases being sinistral multiple, dextral multiple, and bilateral multiple, respectively. Axillary LN L/S ratios were less than or equal to 2 in four cases (57.14%, median: 1.86, IQR: 1.11, range: 1.09–3.9). Five cases (19.23%) had inguinal lymphadenectasis, with one case (3.85%) being dextral multiple and four cases (15.38%) being bilateral multiple. Inguinal LN L/S ratios were less than or equal to 2 in one case (20%, median: 3.5, IQR: 1.13, range: 1.56–6.33). Retroperitoneal lymphadenectasis occurred in 12 cases (46.15%, with 7 cases from CT), and retroperitoneal LN L/S ratios were less than or equal to 2 in three cases (60%, median: 1.92, IQR: 0.55, range: 1.31–2.47).

**Figure 1:**
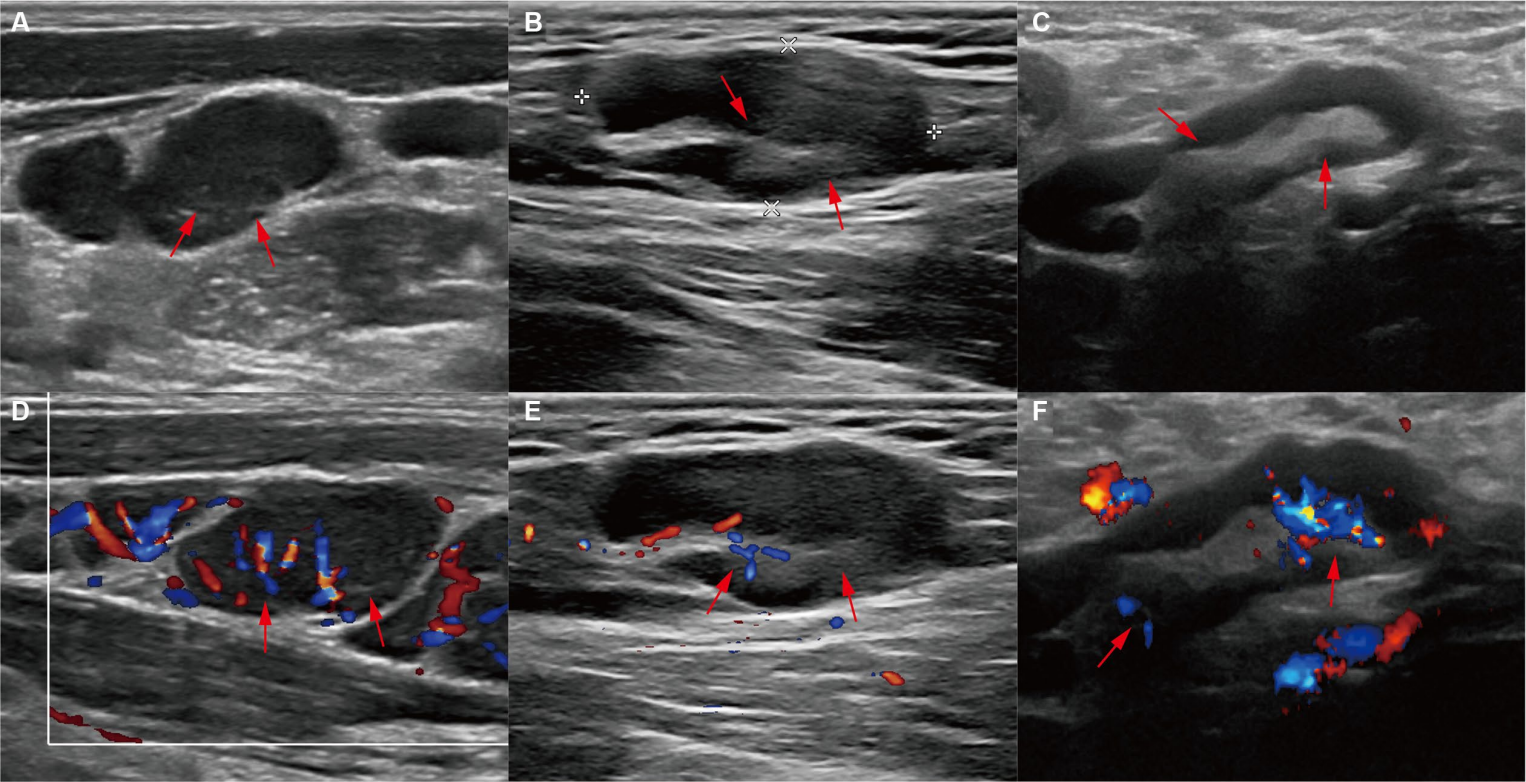
Ultrasound features of present or thinned hila. **(A)** Ultrasound images and **(D)** color Doppler flow imaging (CDFI) of the left cervical lymph node (LN) from a 34-year-old patient showed a thinned hilum and a hilar-type blood flow signal (red arrows). **(B)** Ultrasound images and **(E)** CDFI of the left cervical LN from a 31-year-old patient showed a thinned hilum and a hilar-type blood flow signal (red arrows). **(C)** Ultrasound images and **(D)** CDFI of the left axillary LN from a 53-year-old patient showed a present hilum and a hilar-type blood flow signal (red arrows).

**Figure 2:**
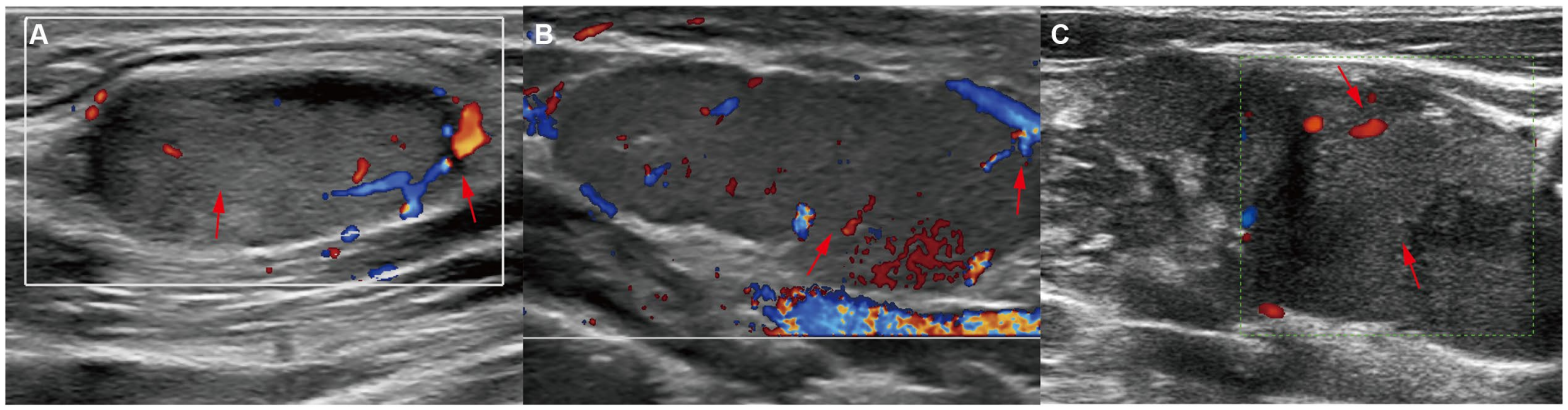
Ultrasound features of absent hila. CDFI of **(A)** left cervical LN from a 29-year-old patient, **(B)** left cervical LN from a 32-year-old patient, and **(C)** right cervical LN from a 37-year-old patient showed the absence of hila and peripheral-type blood flow signals (red arrows).

The blood flow signals were complex and variable: 1 case was mixed (3.85%), 10 cases were peripheral (38.46%), and 13 were hilar (50%). The hilum of LNs was absent, thinned, or present in 12 cases (46.15%), 10 cases (38.46%), and 4 cases (15.38%), respectively. When the hila were present or thinned, the blood flow signals were primarily of the hilar type, whether rich or poor (Figure 1). However, when the hila were absent, the blood flow signals were mostly peripheral type and poor (Figure 2).

**Figure 3:**
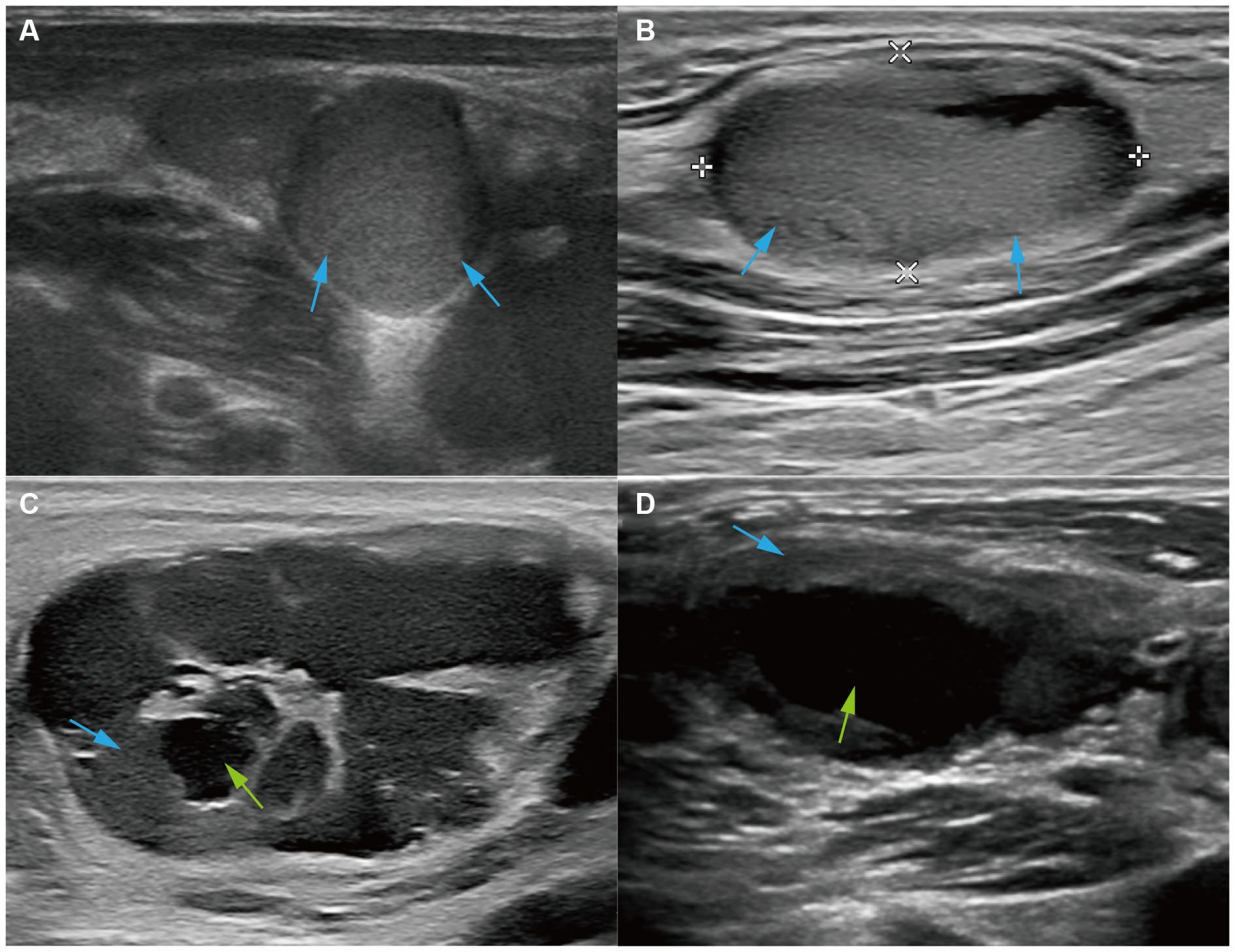
Ultrasound features of echogenicity. Ultrasound images of **(A)** the right cervical LN from a 37-year-old patient and **(B)** the left cervical LN from a 29-year-old patient showed that LNs were hyperechoic (red arrows). Ultrasound images of **(C)** the left cervical LN from a 29-year-old patient and **(D)** the left cervical LN from a 33-year-old patient showed hyperechoic LNs (red arrows) and large liquefaction (green arrows).

For the echogenicity of LNs, 21 cases (80.8%) were hyperechoic; among them, 6 were heterogeneous, and 15 were homogeneous (Figures 3A,B). In three out of five hypoechoic cases, the hila were present, and these pathological expressions were similar to reactive hyperplasia. Six cases (23.08%) showed liquefaction; four out of six cases had less than 15% liquefaction and two out of six cases had more than 50% liquefaction. In particular, all but one case (83.33%) had liquefaction, with their echogenicity being hyperechoic with a tulle-like change (Figures 3C,D).

There was neither fusion nor calcification nor LN sinuses nor obvious edema of peripheral tissue. Given the above findings, the sonographic features of superficial LNs reflected the seriously damaged immune system and rapidly progressing condition of *T.marneffei*-infected HIV patients.

### 3.3 Associations between lymphocyte subsets and sonographic features

To understand the significance of ultrasound examination in determining *T.marneffei* infection, the potential connection between sonographic features of superficial LNs and lymphocyte subsets had been explored. The comparison between the variables was analyzed using the *χ*^2^ test, but there was no significant result. A correlation analysis between LN L/S ratios and the amount of lymphocyte subsets was conducted (Supplementary Figure S2). Axillary LN L/S ratios had a significant positive correlation with CD4^+^T cell counts (*r* = 0.8214, *p* = 0.0341, Supplementary Figure S2A), CD4^+^T cell ratios (*r* = 0.8929, *p* = 0.0123, Supplementary Figure S2C), and CD4^+^T/CD8^+^T cell ratios (*r* = 0.8929, *p* = 0.0123, Supplementary Figure S2B). Inguinal LN L/S ratios also had a high correlation with T cell ratios (*r* = 1, *p* = 0.0167, Supplementary Figure S2D). Cases with different parameter values in Tables 1, 2 were grouped. Patients with retroperitoneal lymphadenectasis had decreased NK cell counts (*p* = 0.03, Supplementary Figure S3A) and NK cell ratios (*p* = 0.0077, Supplementary Figure S3B). These results showed that a lower count of CD4^+^T cells and T cells might have an association with enlarged superficial LNs.

## 4 Discussion

In this retrospective study, a small cohort of 26 HIV patients with enlargement of LNs and *T.marneffei* coinfection were included. All patients underwent ultrasound-guided needle puncture biopsy of superficial LNs, confirming infection with *T.marneffei*. Clinical information, including patient details, sonographic data, and lymphocyte population, and ultrasound images were collected. Through observation and statistical analysis, we characterized the ultrasound features of superficial LNs in HIV patients infected with *T.marneffei* and showed the possible associations between sonographic data and lymphocyte population.

It is worth noting that diagnoses of AIDS-related superficial LN tuberculosis (ASLTB) and AIDS-related diffuse large B cell lymphoma (ADLBL) should be differentiated in cases of *T.marneffei*-related LN infection. Ultrasound findings for ASLTB often show superficial LN enlargement in multiple locations, with the neck being the most common site. The images are complex and variable, often showing hypoechoic signs, thinned or absent hilum structure, and internal liquefaction, which makes it difficult to differentiate from *T.marneffei*-infected LNs (Supplementary Figure S4). However, ASLTB has a specific chronic inflammatory response, and the lesion grows slowly, with an increased probability of edema in the surrounding tissue. There is usually no obvious tenderness upon local compression, and LNs often fuse with visible calcification. Some cases may involve the skin and form sinus tracts. In contrast, *T.marneffei* infection of LNs has a more acute onset and rapid progression, often accompanied by tenderness upon local compression. Adjacent LNs are rarely fused, and skin lesion is rare, with no formation of sinus tracts. When liquefaction occurs, the solid part still shows diffuse echogenicity. Lymphoma is a common AIDS-related malignancy. The internal part of the affected LN shows extremely low echogenicity, which is often lower than that of the surrounding muscle tissue, and typical small mesh-like changes can be noticed. The hilum is basically absent, and calcification is rare. Color Doppler flow imaging (CDFI) often shows a rich portal-type blood flow, with many branches and a few cases showing mixed or marginal-type blood flow (Supplementary Figure S5). The progression is usually slow, and the affected LNs are usually large, with an L/S ratio of less than 2 and a mean diameter of over 5 cm. There is usually no tenderness upon local compression. In CDFI scans of patients with the *T.marneffei* infection, the mean diameter of the involved superficial LNs is rarely over 3 cm. The internal echogenicity is higher than that of ADLBL, and liquefaction is rare. The hilum of LNs may exist or become thinner, and tenderness is often present upon local compression. The type of *T.marneffei*-infected LNs characterized by the absence of diffuse echogenicity increase in the LN and the usually present or thinner hilum is prone to misdiagnosis and relies more on clinical diagnosis. Therefore, sometimes, it is difficult to discriminate by ultrasound alone, and ultrasound-guided puncture biopsy of LNs is often needed for pathological diagnosis.

*Talaromyces marneffei* is a thermally dimorphic fungus primarily prevalent in Southeast Asia and East Asia (24). It usually infects people who have low immunity, especially immunocompromised HIV patients (25). In HIV patients, the quantity of CD4^+^T cell can reflect the body’s immunity because it is the major target cell of HIV. Clinical researchers found that the majority of *T.marneffei*-infected HIV patients had less than 50 CD4^+^T cell counts (26), and it had certain diagnostic efficacy for early AIDS combined with the *T.marneffei* infection (27). The same result was found in this study, with CD4^+^T cell counts less than 200, 100, and 50 cells/μL in 23 (92%), 21 (84%), and 20 (80%) patients, respectively. The correlation analysis between CD4^+^T cell counts, CD4^+^T cell ratios, CD4^+^T/CD8^+^T cell ratios, and axillary LN L/S ratios showed that the quantity of CD4^+^T cell had a significantly positive correlation with axillary LN L/S ratios. Decreased CD4^+^T cells indicated that the immune system was damaged. The LN L/S ratio less than 2 was known as malignant enlargement of LNs (28), so this relationship between CD4^+^T cell and LN L/S ratios may be explained as the worse immune status resulting in a heavier infection. Another subset of blood lymphocytes, NK cells, was reported to be decreased in *T.marneffei*-infected HIV-negative patients (29, 30). Apart from their decrease in HIV patients, lower NK cell counts and NK cell ratios were observed in patients who had retroperitoneal lymphadenectasis. To summarize, similar to lymphocyte subsets such as CD4^+^T cells, ultrasound findings of LNs could serve as indicators for the *T.marneffei* infection in HIV patients.

This study had several limitations, with the major one being the small sample size. The reason only 26 patients were chosen for the study was the inclusion criteria set by us. The present study aimed at exploring the ultrasound characteristics of superficial LNs; only patients confirmed to be infected with *T.marneffei* through ultrasound-guided needle puncture biopsy of superficial LNs, followed by the tissue culture, met the inclusion criteria. This led to a small sample size. Additionally, affected by the limited sample size, the credibility of the results of statistical analysis in this study was reduced. In future research, we might expand the sample size of *T.marneffei*-infected HIV patients with enlarged LNs and collect patients with HIV and other pathogen coinfection, except *T.marneffei*, who also have enlarged LNs into another group. Following this, we might attempt to characterize the differences among patients who have LN enlargement due to infections of different causes.

## 5 Conclusion

In summary, *T.marneffei* infection of LNs is common in HIV patients with a rapid onset and often present with superficial LN enlargement, mostly involving the cervical LNs. The *T.marneffei*-infected LNs have several characteristics in echogenicity, hilum, and blood flow signal, which is different from ALSTB or ADLBL. Furthermore, there might be associations between lymphocyte subsets and enlarged superficial LNs. Ultrasound examination should be paid attention to if patients have superficial LN enlargement and if the diagnosis of *T.marneffei* infection is considered.

## Data availability statement

The raw data supporting the conclusions of this article will be made available by the authors, without undue reservation.

## Ethics statement

The studies involving humans were approved by Clinical Research Ethics Committee of the Hangzhou Xixi Hospital. The studies were conducted in accordance with the local legislation and institutional requirements. The ethics committee/institutional review board waived the requirement of written informed consent for participation from the participants or the participants’ legal guardians/next of kin because This research used data or specimens obtained in previous clinical trials, disease surveillance or clinical studies.

## Author contributions

LP conceived and designed this study and collected the data and images. HS designed this study, analyzed the data and wrote the manuscript. All authors reviewed and approved the final manuscript.
