# Supplementary material for: Ultrasound characterization of superficial lymph nodes in HIV patients with Talaromyces marneffei infection
Source: Front Med (Lausanne). 2023 Dec 11;10:1243599. doi: 10.3389/fmed.2023.1243599 (PMC10750475; doi:10.3389/fmed.2023.1243599)
Supplement: Supplementary file 1 [file Data_Sheet_1.docx]

Supplementary Material

Ultrasound Characterization of Superficial Lymph Nodes in HIV Patients with *Talaromycosis marneffei* Infection

Huaguo Shao, Lin Pan*

*** Correspondence:** Lin Pan: feli0000@163.com.

# Supplementary Figures

## Supplementary Figure 1


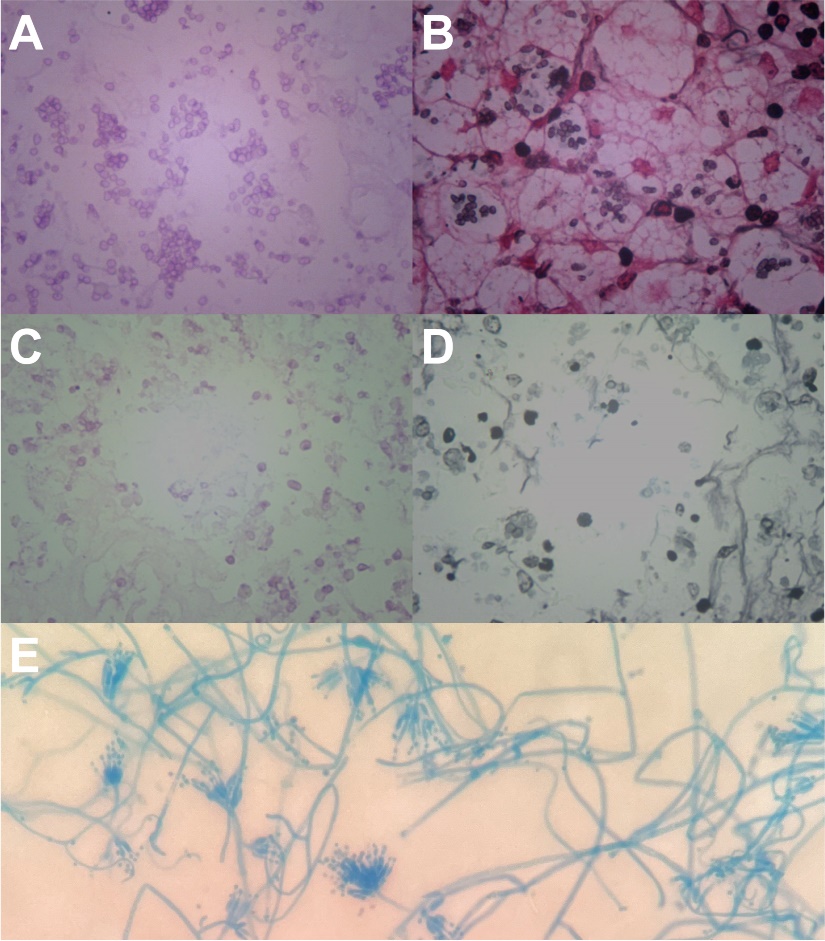


**Supplementary Figure 1. Histopathological staining and fungal culture result of partial patients.**

PAS staining (×400) and (B) GMS staining (×400) of right cervical lymph node biopsy from a 40-year-old patient showed damage of lymphatic tissue and clustered and coagulative necrosis of large numbers of cells which phagocytized spores. (C) PAS staining (×400) and (D) GMS staining (×400) of left cervical lymph node biopsy from a 57-year-old patient showed mass necrosis and the lymphatic tissue is not clear. (E) culture result from a 29-year-old patient showed morphology of *T. marneffei* colonies on Sabouraud agar medium.

## Supplementary Figure 2


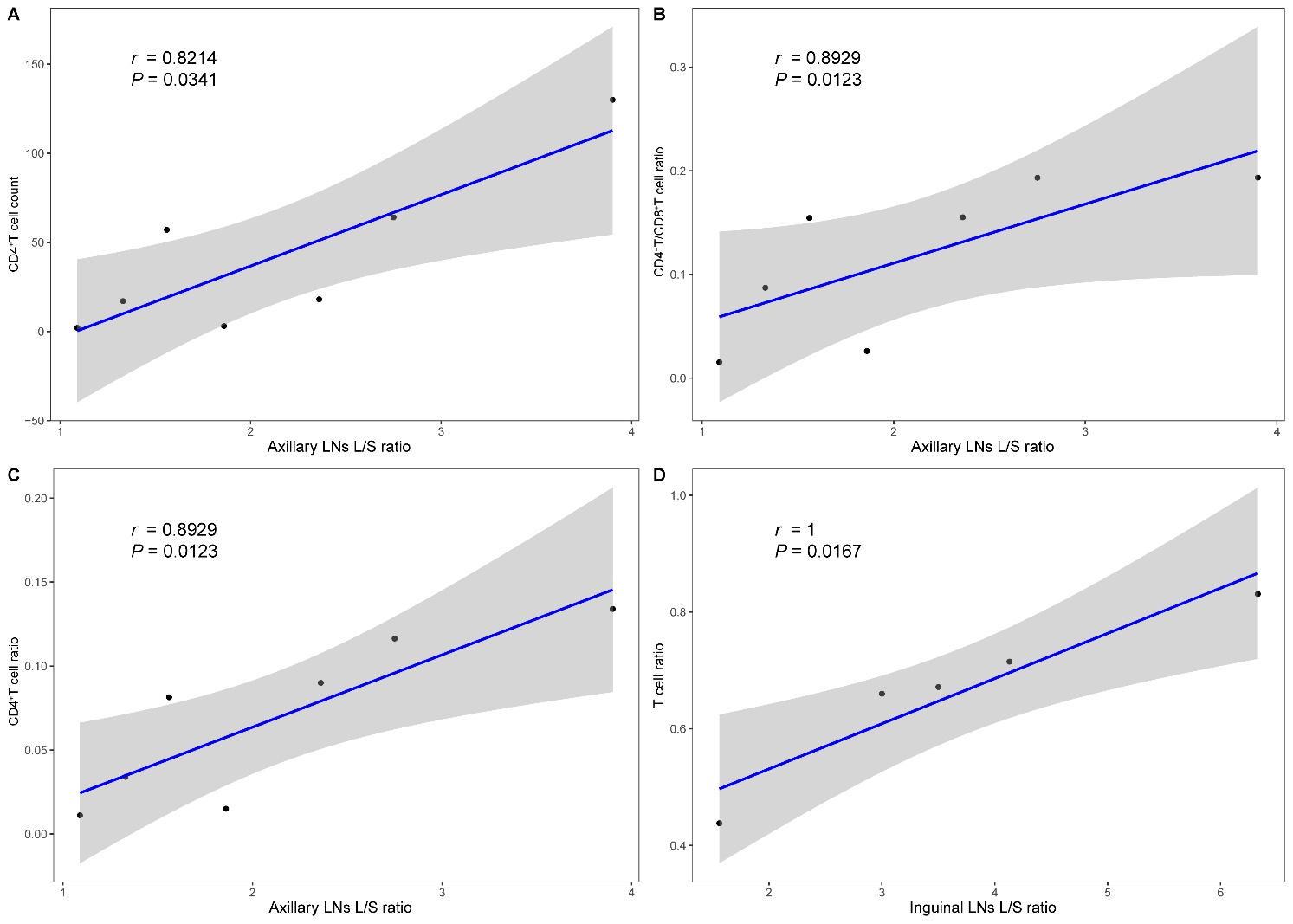


**Supplementary Figure 1.** **Correlation between lymphocyte subsets and LNs L/S ratios**

The correlation analysis of between (A) CD4^+^T cell counts and axillary LNs L/S ratios, (B) CD4^+^/CD8^+^T cell ratios and axillary LNs L/S ratios, (C) CD4^+^T cell ratios and axillary LNs L/S ratios and (D) T cell ratios and inguinal LNs L/S ratios. Each point represents one case. Blue lines were fitted curve function generated by method “lm”. Grey areas were 95% confidence intervals of each fitted curve. The correlation coefficient *r* and *P* value were marked in each plot.

## Supplementary Figure 3


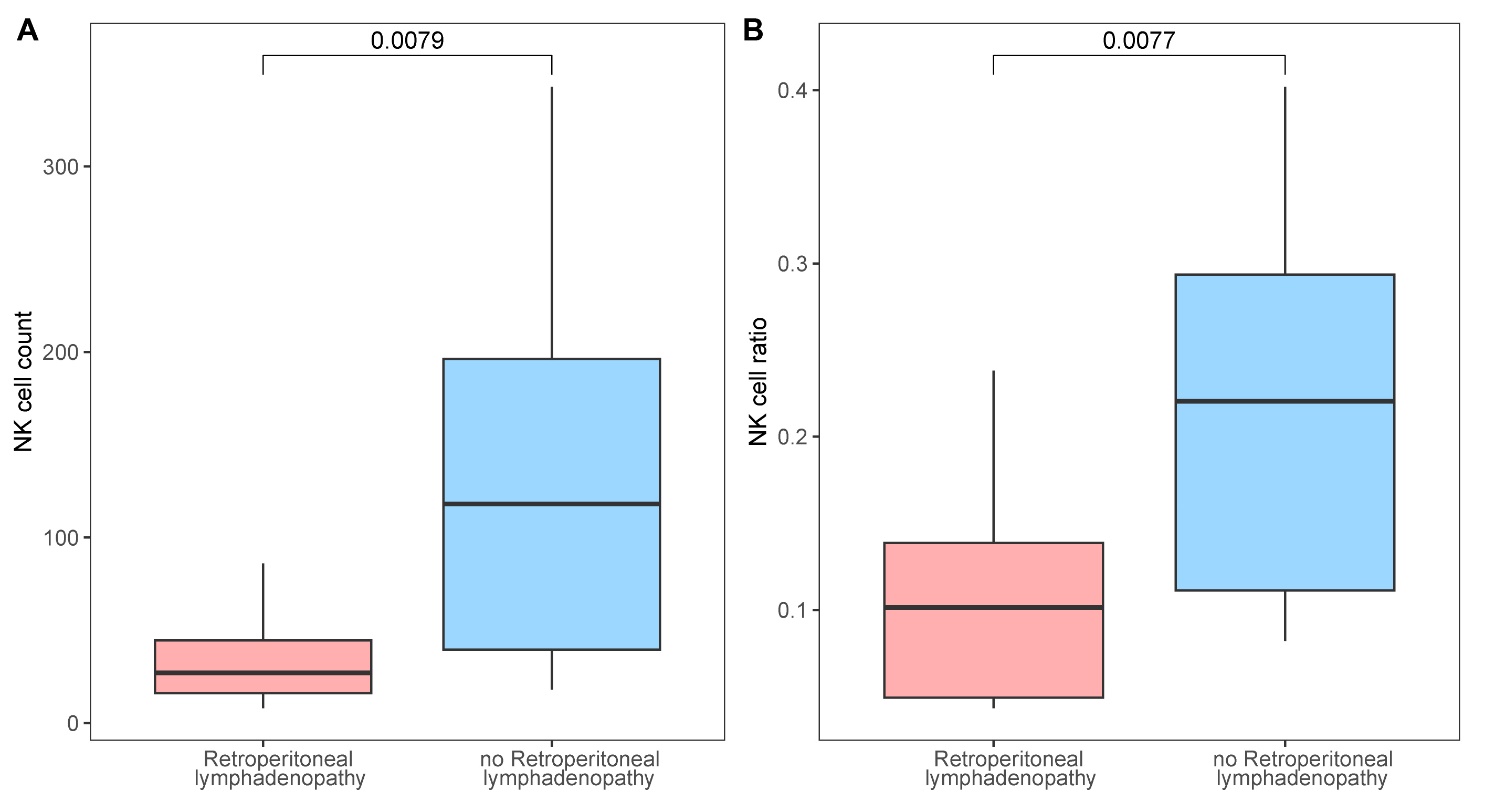


**Supplementary Figure 3. Boxplots of lymphocyte subsets.**

Boxplots of (A) NK cell counts, (B) NK cell ratios between retroperitoneal lymphadenectasis cases and no retroperitoneal lymphadenectasis cases. Differences of groups were analyzed using Mann-Whitney U non-parametric tests. *P* values were marked out above auxiliary lines.

## Supplementary Figure 4


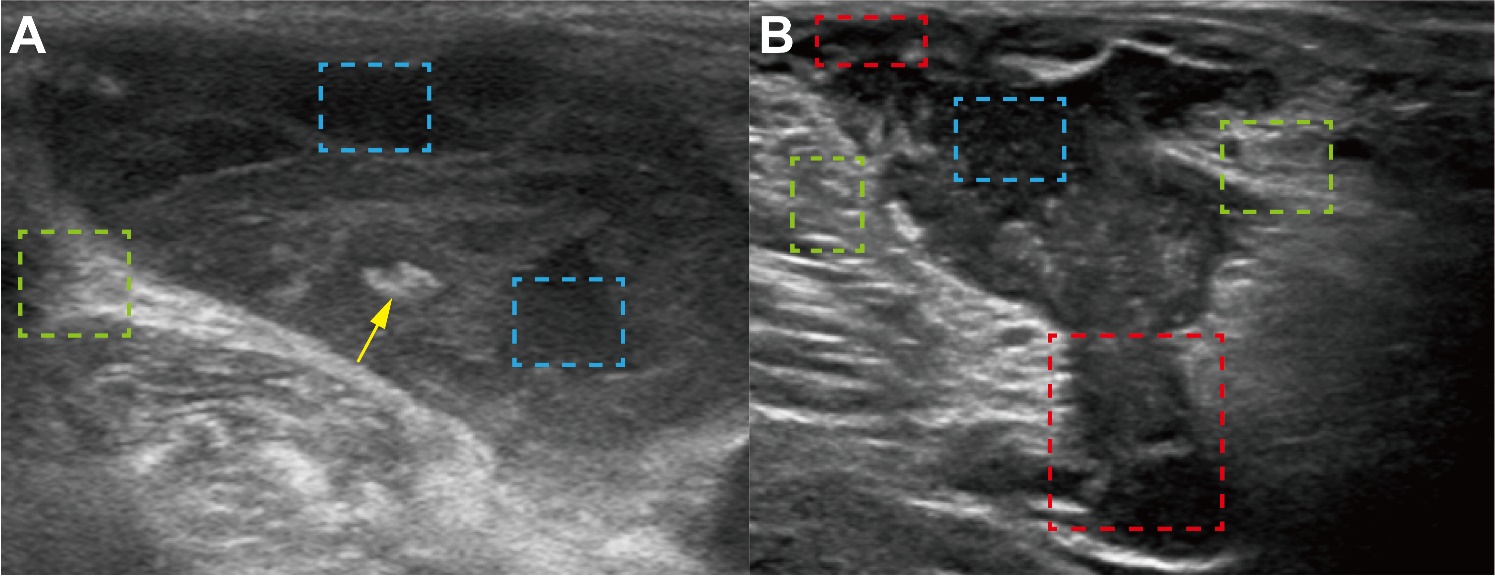


**Supplementary Figure 4. Ultrasound images of right cervical lymph node from a 29-year-old ASLTB patient.**

(A) The interior echo of LNs were hypoechoic, heterogeneous; the hilum was absent; Liquefaction, representing the caseous necrosis (blue dotted box), calcification (yellow arrow) and edema of surrounding soft tissue were observed (green dotted box). (B) Sinus (red dotted box), edema of surrounding soft tissue (green dotted box) and caseous necrosis (blue dotted box) were observed.

## Supplementary Figure 5


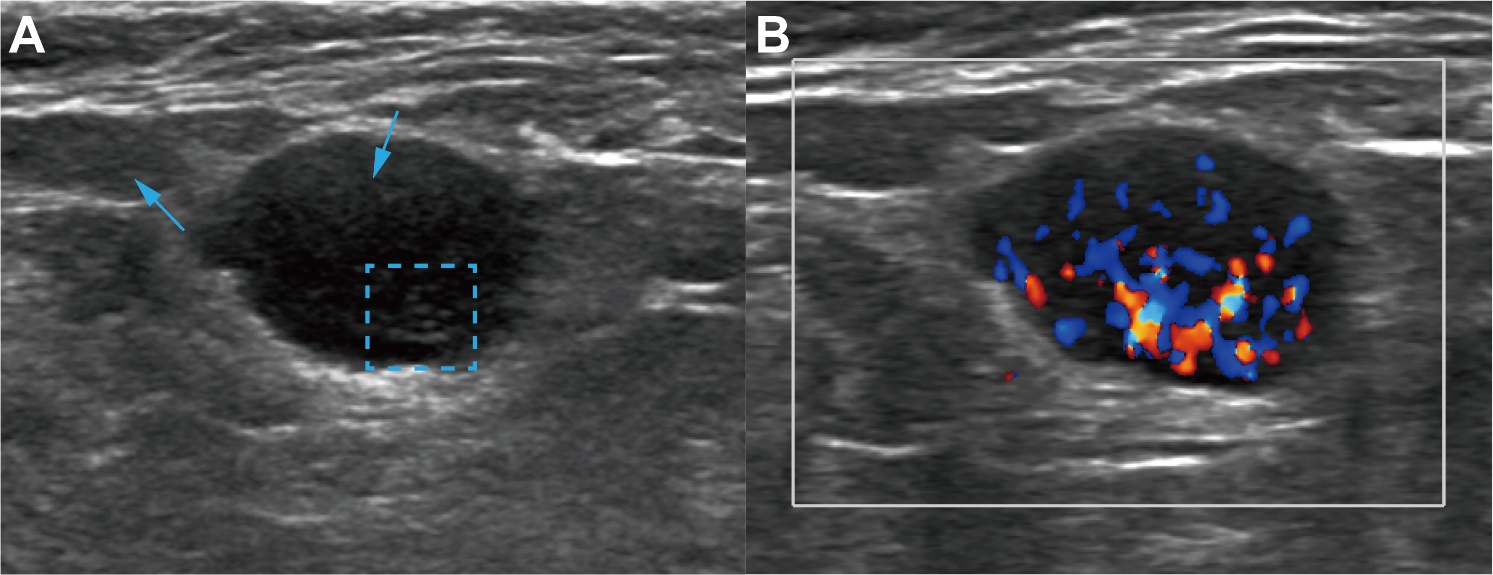


**Supplementary Figure 5. Ultrasound images of left axillary lymph node from a 66-year-old ADLBL patient.**

(A) The interior echo of LNs were hypoechoic, even lower than sounding muscular tissue (blue arrows) and grid-like change was observed (blue dotted box). (B) CDFI showed the blood flow signal was rich and hilar type.
